# Anthropometric and Three-Compartment Body Composition Differences between Super League and Championship Rugby League Players: Considerations for the 2015 Season and Beyond

**DOI:** 10.1371/journal.pone.0133188

**Published:** 2015-07-29

**Authors:** Ben Jones, Kevin Till, Matthew Barlow, Matthew Lees, John Paul O’Hara, Karen Hind

**Affiliations:** Research Institute for Sport, Physical Activity and Leisure, Leeds Beckett University, Leeds, West Yorkshire, United Kingdom; Karolinska Institutet, SWEDEN

## Abstract

Super League (SL) and Championship (RLC) rugby league players will compete against each other in 2015 and beyond. To identify possible discrepancies, this study compared the anthropometric profile and body composition of current SL (full-time professional) and RLC (part-time semi-professional) players using dual-energy X-ray absorptiometry (DXA). A cross-sectional design involved DXA scans on 67 SL (n=29 backs, n=38 forwards) and 46 RLC (n=20 backs, n=26 forwards) players during preseason. A one-way ANOVA was used to compare age, stature, body mass, soft tissue fat percentage, bone mineral content (BMC), total and regional (i.e., arms, legs and trunk) fat and lean mass between SL forwards, SL backs, RLC forwards and RLC backs. No significant differences in age, stature or body mass were observed. SL forwards and backs had relatively less soft tissue fat (17.5 ± 3.7 and 14.8 ± 3.6 *vs*. 21.4 ± 4.3 and 20.8 ± 3.8%), greater BMC (4,528 ± 443 and 4,230 ± 447 *vs*. 4,302 ± 393 and 3,971 ± 280 g), greater trunk lean mass (37.3 ± 3.0 and 35.3 ± 3.8 *vs*. 34.9 ± 32.3 and 32.3 ± 2.6 kg) and less trunk fat mass (8.5 ± 2.7 and 6.2 ± 2.1 *vs*. 10.7 ± 2.8 and 9.5 ± 2.9 kg) than RLC forwards and backs. Observed differences may reflect selection based on favourable physical attributes, or training adaptations. To reduce this discrepancy, some RLC players should reduce fat mass and increase lean mass, which may be of benefit for the 2015 season and beyond.

## Introduction

Rugby league (RL) is a high-intensity, collision team sport that is played at international, professional, amateur and junior levels, mainly within Europe and Australasia. Domestically, the European Super League (SL) and Australasian National Rugby League (NRL) are regarded as the highest playing standard world-wide [1]. In the United Kingdom (UK) and France, prior to the 2015 season, 14 full-time professional clubs competed against each other in the SL and 14 part-time semi-professional clubs competed against each other in the Rugby League Championship (RLC). Further, there was no promotion into or relegation out of the SL, as this was based on a franchise system. However, from the 2015 season, there will be a change to the competition’s structure. This will see promotion into and relegation from the SL reintroduced for the first time in 6 years. The new structure will consist of 12 SL teams competing against each other twice and 12 RLC teams competing against each other twice. Following this, the top 8 SL teams will compete against each other before entering into a play-off system, prior to the winner being determined in a grand final. Of relevance, some SL and RLC teams will compete against each other within a league format for the first time in the competition’s history. The bottom 4 SL teams will play the top 4 RLC teams in a league to decide who competes in SL the following season. Based on league position, the top 3 will return to SL for the following year. Teams positioned 4^th^ and 5^th^ will then compete in a match, with the winner also securing a place in SL the following year.

Success in RL requires players to have well-developed anthropometric (i.e., height and body mass [BM]), body composition and physical (i.e., strength, power, speed, agility and endurance) characteristics [2,3]. BM is regarded as an important characteristic for RL players, because sprint momentum (speed * mass) is a key determinant of playing standard [4], beneficial for ball carrying and defensive situations. However, an increase in BM of 1 kg can increase the aerobic demand of exercise by 1 to 14% [5]. Given the 80 minute duration of RL [2], limiting excess mass (i.e., fat mass) would appear desirable with lower sum of skinfolds related to increased playing minutes [6] and physical performance (i.e., vertical jump, r = -0.345; 30 m sprint, r = 0.417; agility, r = 0.391; aerobic capacity, r = -0.464 [7]). In addition, research supports this, reporting lower skinfolds and greater lean mass in elite compared to semi-elite players [6]. Therefore, excess fat mass could be counterproductive to the power-to-weight ratio, acceleration and metabolic efficiency [8] of RL players.

To date, the majority of research examining the anthropometric and body composition profiles of RL players have used the sum of skinfold assessment to determine body composition [6,9,10,11]. Although skinfold assessments have advantages in relation to time and ease of use, the accuracy and reliability of estimating body fat percentage and lean mass is a limitation [12]. RL players are typically classified into two positional playing groups; forwards and backs [2], with known anthropometric and body composition differences due to the varying demands of match play. RL backs are shorter, lighter, with a lower sum of skinfolds compared to forwards [13,14]. This is beneficial given their greater time engaged in high-intensity running (2.0 ± 0.4 *vs*. 1.4 ± 0.3%), with forwards spending a greater proportion of time in collisions than backs (6.5 ± 0.9 *vs*. 2.7 ± 0.8%) [15]. In comparison to skinfold assessments, a more valid and reliable method of body composition is dual-energy x-ray absorptiometry (DXA), which has recently been used within studies in rugby [12,16,17,18]. DXA evaluations analyse three-compartment body composition, allowing fat mass, lean mass and bone mineral content (BMC) to be calculated, alongside valid and reliable measures of regional body composition [19].

Given the re-structuring of the leagues, it is timely to investigate the differences between SL forwards, SL backs, RLC forwards and RLC backs for stature, BM, and DXA-derived three compartment total and regional body composition. In doing so, valuable avenues might be identified for the physical preparation of players for 2015 and beyond. Given that SL players are full-time professional and RLC players are more likely to be part-time semi-professional, it is hypothesized that SL forwards and RLC forwards are taller and heavier, with greater lean and fat mass than SL backs and RLC backs. It is also hypothesized that SL forwards and SL backs would have greater lean mass and lower fat mass in comparison to RLC forwards and RLC backs.

## Materials and Methods

### Design

A cross-sectional design was used to compare the stature, BM, soft tissue fat percentage, bone mineral content alongside total and combined (i.e., left and right limb / trunk) regional (arms, legs, and trunk) fat mass and lean mass of SL forwards, SL backs, RLC forwards and RLC backs. Players were classified based on their primary playing position. Forwards included props, hookers, second rowers, and loose forwards. Backs included half-backs, centres, wingers, and full-backs. Players were tested during the last phase of the pre-season training period (January–February), two to three weeks prior to their first competitive league match, where body composition would be expected to be optimal.

### Participants

One hundred and thirteen RL players participated in the study. Sixty-seven professional players were from two European SL clubs (backs: *n* = 29, forwards: *n* = 38), and forty-six semi-professional players were from two RLC clubs (backs: *n* = 20 and forwards: *n* = 26). Ethics approval for the study was granted by the Carnegie Faculty Research Ethics Committee (Leeds Beckett University, UK) and complied with the Declaration of Helsinki on human research and international standards. Written informed consent was provided in addition to permission from the respective rugby clubs.

### Anthropometric and DXA measurement and analysis

Stature was measured using a stadiometer (SECA Alpha, Birmingham, UK) to the nearest 0.1 cm and BM was measured using calibrated electronic scales (SECA Alpha 770, Birmingham, UK) to the nearest 0.1 kg. All participants were scanned in a euhydrated state (urine osmolality <700 mOsmol∙kg^-1^ [20]), on a non-training day in the morning. For all measurements, participants wore minimal clothing, with shoes and jewellery removed. Each participant received one total body DXA scan (Lunar iDXA, GE Medical Systems, UK) using standard or thick mode depending on stature and body mass. The mode used was automatically selected by the software and was dependent on body thickness. A body thickness greater than 25 cm indicated thick mode. A body thickness of 25 cm or less indicated standard mode. Participants lay in the supine position on the scanning table with their body aligned with the central horizontal axis. Arms were positioned parallel to the body, with legs fully extended and feet secured with a canvas and Velcro support to avoid foot movement during the scan acquisition.

**Fig 1:**
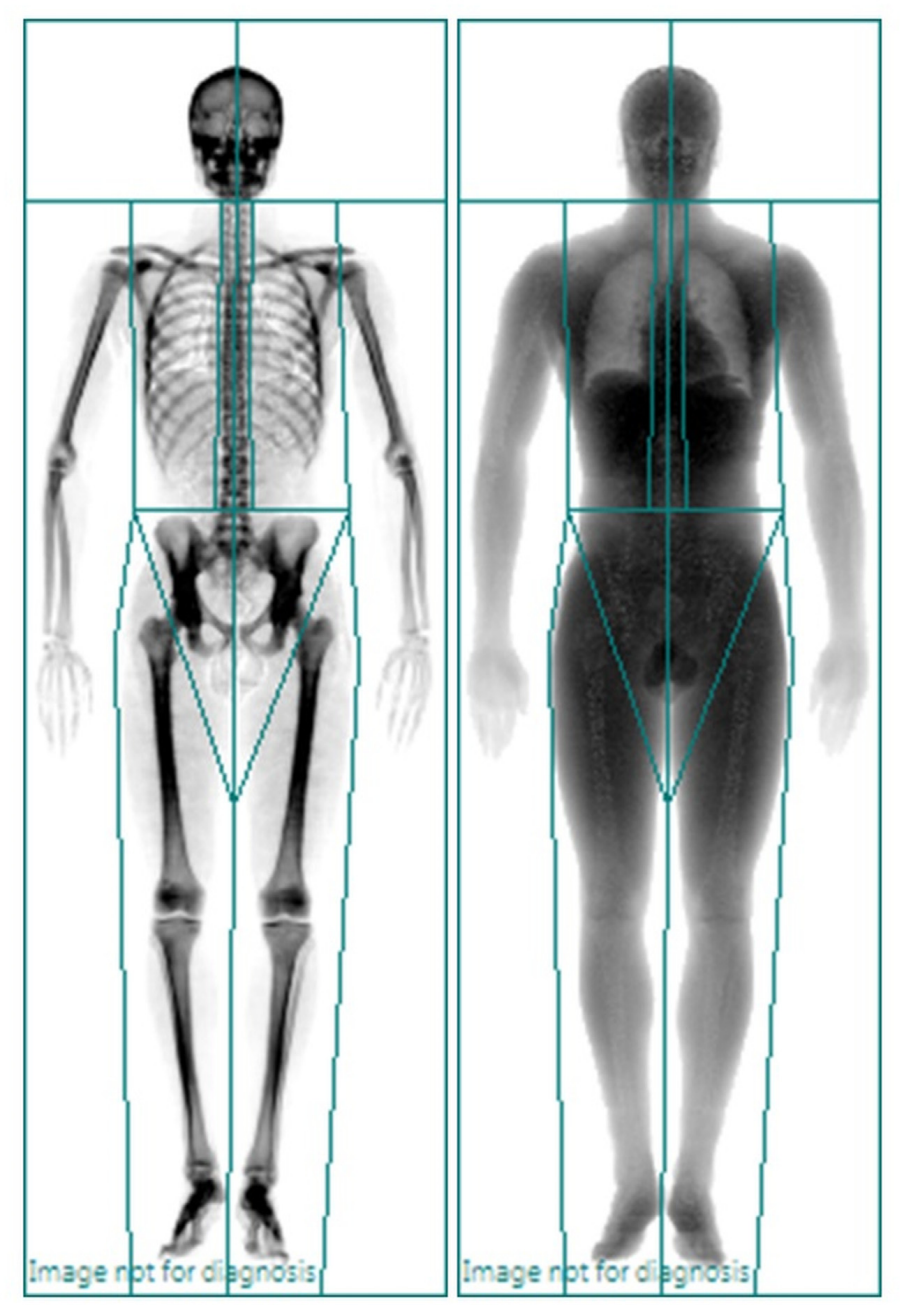
The regions of interest to enable the appropriate cuts according to the manufacturer’s instructions.

One skilled technologist led and analysed all scans following the manufacturer’s guidelines for patient positioning. The regions of interest were manually placed to enable the appropriate cuts according to the manufacturer’s instructions. The regions of interest are illustrated in Fig 1. Defined regions were for the arms, legs and trunk. The appendicular regions of interest for the arms and legs were defined by cut lines positioned proximally at the coracoid process and at the superior iliac crest and lower ramus respectively. The pelvic region was defined through region of interest cuts, through the femoral neck. The trunk region included the pelvis, abdomen and chest. Scan analysis was performed using the Lunar Encore software (Version 15.0). The machine’s calibration was checked and passed on a daily basis using the GE Lunar calibration hydroxyapatite and epoxy resin phantom. There was no significant drift in calibration for the study period. Local precision (test-re-test) values for our Centre (in healthy adult subjects, aged 34.6 years) are 0.8% for total fat mass, 0.5% for total lean mass, and 0.6% for total BMC and bone mineral density (BMD) [21,22]. Regional precision values for our Centre have also been published elsewhere [22].

### Statistical analysis

All statistical analyses were computed using SPSS version 20 (IBM, Armonk, NY, USA), with all statistical significance set at *p*<0.05. Before analysis, normality and equality of variance of the variables were assessed using a Kolmogorov-Smirnov test. One-way ANOVA were used to compare body size and body composition parameters between the SL and RLC backs and forwards. A Bonferroni *post-hoc* analysis was used to determine where any significant differences occurred. SPSS applies the Bonferroni adjustment by multiplying all *p* values for *post-hoc* comparisons by the number of pairwise comparisons (*n* = 6), to allow the alpha level to remain as *p*<0.05. Effect sizes, using Cohen’s *d* were calculated between groups. For Cohen’s *d*, a modification to the effect size scale of Cohen [23] were used and were interpreted as follows: 0–0.19 was considered to be a ‘trivial’ effect, 0.2–0.59 ‘small’, 0.6–1.19 ‘moderate’, 1.2–2.0 ‘large’, and >2.0 ‘very large’ effect [24]. 95% confidence intervals were also calculated. Where the confidence interval crossed 0, the effect was interpreted as unclear [24].

## Results

Table 1 presents the mean ± standard deviation (SD) for age, stature, BM, soft tissue fat percentage, total fat mass, total lean mass and total BMC for SL forwards, SL backs, RLC forwards and RLC backs. Table 2 presents arm, leg and trunk fat and lean mass for SL forwards, SL backs, RLC forwards and RLC backs.

**Table 1:** Anthropometric and total three-compartment body composition of rugby league Super League (SL) and Championship (RLC) forwards and backs.

	Super League		Championship		ANOVA *P*	Post-hoc	*p* = / Cohen’s *d* (95% CI)					
	Fwds (1)	Backs (2)	Fwds (3)	Backs (4)			SL Fwds v SL Backs	SL Fwds v RLC Fwds	SL Fwds v RLC Backs	SL Backs v RLC Fwds	SL Backs v RLC Backs	RLC Fwds v RLC Backs
Age (years)	25.2	24.8	26.1	25.7	0.70		*p* = 1.000	*p* = 1.000	*p* = 1.000	*p* = 1.000	*p* = 1.000	*p* = 1.000
	± 2.9	± 4.3	± 4.9	± 4.3			*d* = 0.11	*d* = -0.22	*d* = -0.14	*d* = -0.28	*d* = -0.21	*d* = 0.09
							(-0.37–0.59)	(-0.73–0.27)	(-0.69–0.40)	(-0.81–0.25)	(-0.78–0.37)	(-0.50–0.67)
Stature (cm)	184.3	181.3	182.3	180.1	0.06		*p* = 0.268	*p* = 1.000	*p* = 0.071	*p* = 1.000	*p* = 1.000	*p* = 1.000
	± 3.2	± 6.1	± 5.9	± 7.1			*d* = 0.62	*d* = 0.42	*d* = 0.76	*d* = -0.17	*d* = 0.18	*d* = 0.34
							(0.14–1.13)	(-0.06–0.94)	(0.29–1.41)	(-0.69–0.37)	(-0.39–0.75)	(-0.25–0.92)
BM (kg)	99.8	90.2	98.4	90.8	<0.001	1, 3 > 2, 4	*p*<0.001	*p* = 1.000	*p* = 0.001	*p* = 0.004	*p* = 1.000	*p* = 0.021
	± 8.1	± 9.1	± 8.4	± 8.7			*d* = 1.11	*d* = 0.17	*d* = 1.07	*d* = -0.94	*d* = -0.07	*d* = 0.89
							(0.59–1.63)	(-0.33–0.67)	(0.49–1.64)	(-1.48– -0.36)	(-0.64–0.50)	(0.27–1.49)
Soft tissue fat (%)	17.5	14.8	21.4	20.8	<0.001	2 < 1< 3, 4	*p* = 0.027	*p* = 0.001	*p* = 0.016	*p<*0.001	*p*<0.001	*p* = 1.000
	± 3.7	± 3.6	± 4.3	± 3.8			*d* = 0.74	*d* = -0.97	*d* = -0.88	*d* = -1.66	*d* = -1.62	*d* = 0.15
							(0.23–1.23)	(-1.50– -0.45)	(-1.44– -0.31)	(-2.26– -1.04)	(-2.26– -0.95)	(-0.44–0.73)
Total fat mass (kg)	16.8	12.7	20.1	18.2	<0.001	2 < 1, 3, 4;	*p* = 0.001	*p* = 0.011	*p* = 1.000	*p*<0.001	*p*<0.001	*p* = 0.697
	± 4.2	± 3.4	± 4.4	± 4.5		1 < 3	*d* = 1.07	*d* = -0.77	*d* = -0.87	*d* = -1.88	*d* = -1.38	*d* = 0.43
							(0.53–1.56)	(-1.28–0.25)	(-0.31–0.22)	(-0.25– -1.23)	(-2.03– -0.76)	(-0.17–1.01)
Total lean mass (kg)	78.5	73.2	73.9	68.6	<0.001	1 > 2, 4	*p* = 0.017	*p* = 0.072	*p<*0.001	*p* = 1.000	*p* = 0.149	*p* = 0.069
	± 6.4	± 7.9	± 7.6	± 5.7			*d* = 0.74	*d* = 0.65	*d* = 1.63	*d* = -0.09	*d* = 0.67	*d* = 0.79
							(0.24–1.24)	(0.15–1.17)	(0.97–2.20)	(-0.62–0.44)	(0.05–1.22)	(0.16–1.36)
Total BMC (g)	4528	4230	4302	3971	<0.001	1 > 2, 4;	*p* = 0.023	*p* = 0.192	*p<*0.001	*p* = 1.000	*p* = 0.186	*p* = 0.045
	± 443	± 447	± 393	± 280		3 > 4	*d* = 0.67	*d* = 0.54	*d* = 1.50	*d* = -0.17	*d* = 0.69	*d* = 0.97
							(0.17–1.16)	(0.02–1.03)	(0.79–1.99)	(-0.70–0.36)	(0.07–1.24)	(0.32–1.55)

Data are presented as mean ± standard deviation. The numbers in parentheses in column headings relate to the numbers used for illustrating significant (*p*<0.05) differences in the *post-hoc* analysis between groups. *Post-hoc p* values have Bonferroni adjustments applied. To allow the alpha level to remain as *p*<0.05, all *p* values for *post-hoc* comparisons are multiplied by the number of pairwise comparisons (*n* = 6) by the statistical package software. BMC = bone mineral content, BM = body mass.

**Table 2:** Regional body composition profiles of rugby league Super League (SL) and Championship (RLC) forwards and backs.

	Super League		Championship		ANOVA *P*	Post-hoc	*p* = / Cohen’s *d* (95% CI)					
	Fwds (1)	Backs (2)	Fwds (3)	Backs (4)			SL Fwds v SL Backs	SL Fwds v RLC Fwds	SL Fwds v RLC Backs	SL Backs v RLC Fwds	SL Backs v RLC Backs	RLC Fwds v RLC Backs
Arm fat mass (kg)	1.8	1.4	2.1	1.9	<0.001	2 < 1, 3, 4	*p* = 0.004	*p* = 0.071	*p* = 1.000	*p*<0.001	*p* = 0.002	*p* = 0.837
	± 0.5	± 0.3	± 0.5	± 0.5			*d* = 0.97	*d* = -0.60	*d* = -0.20	*d* = -1.70	*d* = -1.21	*d* = 0.40
							(0.42–1.44)	(-1.10– -0.08)	(-0.74–0.35)	(-2.31– -1.08)	(-1.87– -0.63)	(-0.20–0.98)
Leg fat mass (kg)	5.6	4.3	6.4	5.9	<0.001	2 < 1, 3, 4	*p* = 0.002	*p* = 0.159	*p* = 1.000	*p*<0.001	*p* = 0.001	*p* = 1.000
	± 1.3	± 1.2	± 1.7	± 1.6			*d* = 1.04	*d* = -0.53	*d* = -0.21	*d* = -1.43	*d* = -1.13	*d* = 0.30
							(0.51–1.53)	(-1.04– -0.03)	(-0.75–0.33)	(-2.01– -0.83)	(-1.76– -0.53)	(-0.29–0.88)
Leg lean mass (kg)	26.8	24.8	25.5	23.8	<0.001	1 > 2, 4	*p* = 0.026	*p* = 0.379	*p* = 0.001	*p* = 1.000	*p* = 1.000	*p* = 0.264
	± 2.6	± 3.1	± 3.2	± 2.3			*d* = 0.70	*d* = 0.45	*d* = 1.22	*d* = -0.22	*d* = 0.37	*d* = 0.61
							(0.20–1.20)	(-0.06–0.95)	(0.60–1.77)	(-0.75–0.31)	(-0.22–0.92)	(-0.01–1.18)
Trunk fat mass (kg)	8.5	6.2	10.7	9.5	<0.001	2 < 1, 3, 4.	*p* = 0.003	*p* = 0.007	*p* = 0.851	*p*<0.001	*p*<0.001	*p* = 0.838
	± 2.7	± 2.1	± 2.8	± 2.9		1 < 3	*d* = 0.95	*d* = -0.80	*d* = -0.36	*d* = -1.82	*d* = -1.30	*d* = 0.42
							(0.42–1.43)	(-1.31– -0.28)	(-0.90–0.19)	(-2.43– -1.18)	(-1.95– -0.70)	(-0.17–1.00)
Trunk lean mass (kg)	37.3	35.3	34.9	32.3	<0.001	4 < 1, 2.	*p* = 0.074	*p* = 0.025	*p*<0.001	*p* = 1.000	*p* = 0.013	*p* = 0.052
	± 3.0	± 3.8	± 3.4	± 2.6		1 > 3	*d* = 0.58	*d* = 0.75	*d* = 1.78	*d* = 0.11	*d* = 0.92	*d* = 0.86
							(0.09–1.08)	(0.23–1.26)	(1.09–2.34)	(-0.42–0.64)	(0.28–1.47)	(0.22–1.44)

Data are presented as mean ± standard deviation. The numbers in parentheses in column headings relate to the numbers used for illustrating significant (*p*<0.05) differences in the *post-hoc* analysis between groups. *Post-hoc p* values have Bonferroni adjustments applied. To allow the alpha level to remain as *p*<0.05, all *p* values for *post-hoc* comparisons are multiplied by the number of pairwise comparisons (*n* = 6) by the statistical package software.

### Age, stature and BM

There were no significant differences between groups for age or stature. BM was significantly greater in forwards than backs within and between leagues. There were no significant differences in BM between SL forwards and RLC forwards, or SL backs and RLC backs. Moderate effects for stature were observed between SL forwards and SL backs, and SL forwards and RLC backs. Body mass differences were moderate between SL forwards and SL backs, SL forwards and RLC backs, SL backs and RLC forwards, and RLC forwards and RLC backs.

### Total and regional body fat

SL backs had a significantly lower soft tissue fat percentage compared to all other groups, and soft tissue fat percentage was lower in SL forwards than RLC forwards and RLC backs. Total and regional (arms, legs and trunk) fat mass were significantly lower in SL backs than all other groups. SL forwards had a significantly lower total and regional trunk fat mass than RLC forwards.

Soft tissue fat percentage and total fat mass showed moderate effects between SL forwards and SL backs, SL forwards and RLC forwards, and SL forwards and RLC backs, while large effects were observed between SL backs and RLC forwards, and SL and RLC backs.

A moderate difference was observed for arms and legs fat mass between SL forwards and SL backs. Large effects were observed between SL backs and RLC forwards, and SL backs and RLC backs for arms, legs and trunk fat mass. Differences in trunk fat mass were moderate for SL forwards and SL backs, SL forwards and RLC forwards.

### Total and regional lean mass

SL forwards had a significantly greater total and regional arm and leg lean mass than SL backs and RLC backs. SL forwards and SL backs had significantly greater regional trunk lean mass than RLC backs, and SL forwards had significantly greater regional trunk lean mass than RLC forwards.

Moderate differences were observed between SL forwards and SL backs, SL forwards and RLC forwards, SL backs and RLC backs, and RLC forwards and RLC backs for total lean mass. Differences between SL forwards and RLC backs were large.

Large effects were observed between SL forwards and RLC backs for arms, legs and trunk lean mass, while moderate effects were observed between RLC forwards and RLC backs for arm and trunk lean mass. Moderate effects were observed between SL forwards and SL backs, and SL forwards and RLC forwards for arms lean mass. Moderate effects were also observed between SL forwards and SL backs for legs lean mass, and SL forwards and RLC forwards, and SL backs and RLC backs for trunk lean mass. Small effects were also observed between SL forwards and SL backs for trunk lean mass.

### Total BMC

Corresponding with their greater BM, total BMC was significantly greater in SL forwards than SL backs and RLC backs. Similarly, RLC forwards had a significantly greater total BMC than RLC backs. A small difference was observed between SL forwards and RLC forwards for total BMC. Differences between SL forwards and SL backs, SL backs and RLC backs, and RLC forwards and RLC backs were moderate, while differences between SL forwards and RLC backs were large for total BMC.

## Discussion

This is the first study to compare the anthropometry and three-compartment total and regional body composition parameters of SL and RLC players, using DXA. Our findings suggest that stature and BM are well-matched between SL and RLC forwards and SL and RLC backs. However, there were distinct differences in body composition. Namely these differences are characterised by lower soft tissue fat percentage in SL backs compared to RLC forwards and backs, and greater lean mass in SL forwards than RLC backs, thus we accept our hypothesis. As expected, forwards at both SL and RLC standard were heavier than SL and RLC backs, respectively. The findings of this study identify body composition targets for RLC players competing against SL players in the 2015 league restructure.

Our results demonstrate BM was similar between SL and RLC forwards, and between SL and RLC backs. Although, when observations of body composition were made, the higher fat mass and lower lean mass in RLC forwards might be a disadvantage when the two leagues compete against each other. RL forwards are required to produce high amounts of force whilst withstanding multiple collisions [25,26], thus in addition to BM, the determinants of BM should be considered. BM consisting of a greater amount of lean mass would theoretically allow a higher amount of force production, compared to a BM consisting of a high amount of fat mass. In line with Newton’s second law of motion (F = m·a), the ability to accelerate (a) with a high mass (m), would result in a high amount of force (F) to be applied in collisions [18]. Without the ability to quantify the differences in the acceleration ability of SL and RLC forwards, this notion is a theoretical construct, requiring exploration within a practical setting. Further, the impact of such body composition discrepancies on traumatic injury risk also requires investigation.

The differences in lean and fat mass between SL and RLC backs is of interest, given that backs are required to perform repeated bouts of high-speed running over greater distances than forwards during a match [2]. As such, and given the effect of body fat on performance and the aerobic demand of exercise [5], the greater body fat identified in RLC backs may therefore be a disadvantage in comparison to SL backs. Both examples regarding collisions for forwards and high-speed running for backs apply to the other positional group, given backs are also involved in collisions and forwards are also involved in repeated high-speed running, although it is accepted this is not their primary role in a match.

The explanation for the observed differences is likely due to SL players being full-time professional athletes, whereas RLC players are mainly employed part-time. In this respect, SL players are more likely to be able to dedicate a greater amount of time to training, recovery and nutritional habits, which concurrently would affect lean and fat mass. Further, expertise (i.e., strength and conditioning coaches / nutritionist) are more likely to be available within a professional club as opposed to a semi-professional part-time club. The identified differences in body composition between standards might also exist based on identification, selection and recruitment of players. A recent retrospective study of UK Academy RL players showed that a lower sum of four skinfolds (at 13–15 years old) was associated with long-term career progression [3]. It is likely that a combination of recruitment and training factors contribute to a more favourable body composition profile in SL players.

The main regional differences observed in this study were greater fat and lower lean mass in the trunk of RLC forwards, and greater fat mass in the arms, trunk and legs of RLC backs, compared to their SL peers by position. These differences may reflect training and recovery practices, and suggest that strength and conditioning programmes for RLC players may benefit from focussing on trunk exercises (i.e., whole-body [compound] resistance training exercises, rather than limbs [i.e., arms and legs]), which would develop the trunk more favourably. Optimising trunk composition and strength is key for forwards and backs, creating a stable framework for generating and transferring force during contact [27].

It is also important to consider differences between playing standards and positions (e.g., SL forwards *vs*. RLC backs), because backs need to defend or attack against forwards within a match. The large differences in mean total lean mass between SL forwards and RLC backs (i.e., approx. 10 kg) was far greater than the difference observed between SL backs and RLC forwards (i.e., approx. 1 kg), which may affect a tackle or successful attack, although the technical elements should also be considered. The discrepancy might also increase injury risk amongst RLC players, which has recently been identified in both SL and RLC players [28]. Bone-related injury risk might also be influenced by the lower BMC in RLC players compared to SL players, representing an important avenue requiring prospective research. BMC differences between SL and RLC players may reflect adaptation to loading of the skeleton generated through training and competition [29].

In addition to anthropometry and body composition, further considerations for team success in the new league structure should be given to the skill level [6,30], physical qualities [4,31], and also the collective experience of a team [32]. Further research should seek to identify any possible differences in these attributes. In addition to observations of body composition alone, this study is not without its limitations. DXA measurements are influenced by food and fluid intake as well as prior physical activity. In this study prior physical activity was controlled, although food and fluid intake was not. Food and fluid intake can cause a substantial increase in the typical error of DXA estimates of total and regional lean mass, thus potentially over or under reporting [19]. As such, future studies should look to further standardise body composition assessments when using DXA. In addition, despite this study classifying players by their primary positional groups (i.e., forwards and backs), with an increased sample size, further classification is possible for backs (full-back, winger, centre and half-back) and forwards (prop, hooker, second-row and loose forward), as each position has a unique role within the team. Also, future studies should look to establish and report the ethnic background of players which may affect body composition, in addition to increasing the number of clubs whereby participants were recruited from may reduce any potential recruitment / playing style biased.

## Conclusion

To conclude, body size was well-matched between SL and RLC forwards, and SL and RLC backs, although distinct differences in body composition exist. Specific to the findings of the current study, RLC forwards and backs might benefit from reducing overall fat mass and increasing lean mass, specifically in the trunk in order to match the body composition of players at a higher standard. RLC backs may benefit from reducing fat mass in the limbs. The effects of body composition discrepancies between teams on injury occurrence represent a valuable avenue for further research. An individualised approach should be adopted to optimise the body composition profiles of players in line with on-field performance indicators. In addition to the skill level and collective experience of a team, a greater lean mass and lower body fat for some players might be of value for consideration by RLC teams if they are to physically match SL teams following the 2015 RL league restructure.
